# Plasticity of Innate Lymphoid Cells in Cancer

**DOI:** 10.3389/fimmu-13-886520

**Published:** 2022-05-19

**Authors:** Bernd Heinrich, Firouzeh Korangy

**Affiliations:** ^1^Thoracic and Gastrointestinal Malignancies Branch, Center for Cancer Research, National Cancer Institute, National Institutes of Health, Bethesda, MD, United States; ^2^Department of Gastroenterology, Hepatology and Endocrinology, Hannover Medical School, Hannover, Germany

**Keywords:** innate immunity, plasticity, tumor microenvironment, immunotherapy, cancer

## Abstract

Innate lymphoid cells (ILCs) are a heterogenous population of the innate immune system, enriched at mucosal surfaces and are pivotal regulators of immune homeostasis. ILCs are the innate counterpart of T cells. Like T cells, ILC subsets are highly plastic with their composition and function controlled by alterations in their microenvironment. This plasticity allows for the trans-differentiation between the subsets to rapidly respond to their immune environment. The tumor microenvironment (TME) is a heterogeneous milieu characterized by different cytokines and growth factors. Through interaction with the tumor microenvironment, ILCs can transdifferentiate into different subsets resulting in pro or anti-tumor immunity. Thus, studying ILC plasticity might result in new therapeutic approaches for cancer therapy. In this review, we summarize current findings of the functional and plastic heterogeneity of ILCs in homeostasis as well as disease settings with a specific focus on cancer. We specifically highlight tumor-driven plasticity and how ILC-induced inflammation can impact the tumor microenvironment and anti-tumor immunity.

## Introduction

Innate lymphoid cells (ILCs) are a heterogenous cell population of the innate immune system that regulate inflammation, tissue repair, barrier homeostasis and immune tolerance (1–4). ILCs respond to a complex network of cytokines, alarmins, hormones, neuropeptides, and microbial pathogens in their microenvironment which control ILC presence, phenotype and function (5). ILCs can be found in both lymphoid and non-lymphoid tissues and are mostly concentrated at barrier sites such as mucosal surfaces of gastrointestinal tract, and pulmonary tract.

All ILCs are recombination activating gene (RAG) independent and lack the expression of surface markers defining other immune cells, therefore known as “lineage” negative cells (1, 6).

ILC subsets are derived from a common lymphoid progenitor (CLP) which differentiate into common innate lymphoid precursors (CLIPS) finally developing into either common helper innate lymphoid precursors (CHILPS) or NK precursors (NKPs, differentiating towards NK cells). CHILPS can give rise to ILC precursors and eventually ILC1s, ILC2s and ILC3s (7, 8).

Based on the expression of transcription factors and cytokine signatures that resemble the T helper (Th) cell subsets, ILCs can be classified into 5 groups: cytotoxic natural killer (NK) cells, ILC1, ILC2, ILC3 and lymphoid tissue inducer (LTi) cells (9). Non-cytotoxic helper ILCs are defined by expression of the interleukin (IL)-7 receptor-α chain (CD127), which is not expressed by mature NK-cells (2, 4).

Although ILCs are mainly tissue resident and have been studied in tonsils, intestine, liver, lung, and adipose tissue (10, 11), it is becoming clear that they can traffic to different tissues heavily influenced by environmental signals which affects their plasticity as well as developmental pathway. Several studies have shown the influence of the local tissue environment on the composition of the ILC subsets in inflammatory diseases such as in lungs of patients with chronic obstructive pulmonary disease (COPD) (12) or intestinal mucosa of patients with Crohn’s disease (13). In the context of cancer, ILCs can have both pro-tumor and anti-tumorigenic properties based on their phenotype, location, and the tumor microenvironment. NK cells are part of group 1 ILCs and their role in tumor immunity has been the focus of numerous studies. However, their full potential in immunotherapy has not been unleashed which might be due to plastic transition of NK cells into less cytotoxic ILC1s in the tumor microenvironment (14). Limited data is accumulating on ILC2s and ILC3s in the context of cancer, but their role seems to be ambiguous, having either pro or anti-tumor effects influenced by the surrounding tumor environment. Promising immunotherapy concepts rely on comprehensive knowledge of the facets of the immune system in the tumor microenvironment. Here we focus on the role of ILCs in tumor as well as their plasticity as influenced by tumor and surrounding cells.

## Definition of ILC Subsets

Group 1 ILCs can be further subdivided into two subsets, EOMES^+^NK cells, a counterpart to cytotoxic T cells, and EOMES^-^ helper ILC1s. ILC1 cells express Tbx21 (encoding the T-box transcription factor) and produce interferon (IFN)-γ parallel to Th1 cells (10). NK cells are cytotoxic with high expression of perforin and granzymes, rely on T-bet and EOMES for their differentiation and play a role in direct killing of tumors and virus-infected cells. NK cells have a different developmental pathway than ILC1s (15).

ILC1s have been shown to play a role in early protection against viruses and infection (16, 17). In mice, ILC1s are defined as Lin^-^NK1.1^+^NKp46^+^ CD49a^+^CD49b^-^, do not express EOMES, produce high levels of IFN-γ, TNFSF10 (tumor necrosis factor ligand superfamily member), granzymes and are dependent on T-bet and Hobbit (ZNF683) for their development (18–20). ILC1s were first identified in mouse liver but later also described in intestines, thymus, spleen, lymph nodes, kidney, and salivary glands (21). Human ILC1s have been explored in both lymphoid and non-lymphoid organs; liver, intestine, tonsil, spleen, adipose tissue, skin, blood, and lungs (22).

ILC2s express the transcription factor GATA-3 essential for their differentiation and maturation (23). They secrete IL-4, IL-5, IL-9, IL-13 and promote type 2 immune responses important in allergies, infection and tissue repair during viral infection (24–26). They have been found in different tissues such as adipose tissue, lung, gut, liver, skin, and adipose tissue (22). Human ILC2s express CRTH2 (chemoattractant receptor-homologous molecule expressed on Th2 cells) and NKp30 (Natural killer cell p30 related protein) which influences their activation as well as migration (27). ILC2s also express KLRG1, the inhibitory receptor killer cell lectin-like receptor subfamily G member 1 (KLRG1) which plays a role in ILC2 expansion and cytokine secretion (28).

ILC3s express retinoic acid receptor-related orphan receptor -γt (RORγt, encoded by RORC) and surface marker CD117 (c-kit) and aryl hydrocarbon receptor (AHR) (29). Group 3 ILCs include CCR6^+^ cells also known as lymphoid-tissue-inducer (LTi) cells and CCR6^-^ ILC3 cells. LTi express RORγt and can secrete IL-22 and/or IL-17 and lymphotoxin (30).

ILC3s express CD117(c-kit) and are further divided into subpopulations based on the expression of NCR (natural cytotoxicity receptor), NKp46, NKP44 and NKp30. NKp44^+^ILC3 cells are mainly found in mucosal tissues and secrete IL-22 and IL-17, resembling Th17 and Th22 helper T cells maintaining homeostasis in the mucosal tissue of the intestine (31).

## ILC Plasticity

The term immune cell plasticity can be defined as the ability of the immune cells to change their phenotype as a mechanism to adapt to their dynamic tissue environment. Plasticity of immune cells is crucial in shaping immune responses to different pathological conditions and stimuli. This has been seen in development and differentiation of different immune cell subsets including Th subsets from naïve CD4^+^ T cells (32) as well as in macrophages and neutrophils (33).

Emerging studies show that the initial classification of ILCs into five subsets has been an oversimplification, and a more complex picture of heterogeneity within ILCs is developing (34, 35). As opposed to T cells, ILCs don’t express a TCR, and their function and phenotype is shaped in response to the cytokines in their environment. ILCs express various cytokine and cell surface receptors themselves which can respond to cytokines secreted by other immune cells such as macrophages and dendritic cells enabling them to detect changes in their microenvironment (34, 36). ILC1s express IL-15R, IL-18R, IL-12R, NKp46, NKp44 as well as CD226. ILC2s express IL-9R, IL-2Rα, IL17RB, IL33R, Thymic Stromal Lymphopoietin receptor (TSLPR), Neuromedin U Receptor 1 (NMUR1) and CRTH2. ILC3s express IL-23R, IL-1R, IL-18R, NKp44 and NKp46 (35, 37). ILC plasticity and conversion into other subsets is pivotal to shape their response to different types of pathogenic stimuli in their environment (38).

From conversion of ILC3 and ILC2 cells into IFN-γ secreting ILC1 cells in pathogenesis of gut mucosal inflammation (13) to transformation of ILC2 into ILC3 resulting in inflammation in psoriasis and asthma, ILC plasticity plays a dynamic and essential role in shaping the immune response (39, 40). With the dawn of the new technologies such as single cell RNA-seq (scRNAseq), assay for Transposase-Accessible Chromatin using sequencing (ATAC-seq) and mass cytometry by time-of-flight (CYTOF) to name a few, a new picture of heterogeneity in ILC function and phenotype in different tissues has emerged (41–44). These studies have identified intermediate cell populations and subsets between NK cells and helper ILC1s as well as amongst the other ILC subsets (14, 45–49). ScRNAseq from mouse intestinal lamina propria showed 15 ILC subclusters under homeostatic conditions. These subclusters included all the ILC groups as well as two transcriptional clusters that were not assigned to any subset-specific lineage (42) showing the high level of heterogeneity within the ILCs. Similarly, transcriptional analysis of CD127^+^ human ILCs from tonsil using RNA-seq characterized not only the main ILC1, ILC2 and ILC3 populations, but also three distinct ILC3 subsets, naïve, IL-22 producing and major histocompatibility complex (MHC) class II^+^ (HLA-DR^+^) ILC3 cells (41). Studying the function of these subsets has helped in understanding the role of these subsets under different settings and identified intermediate states which are mediated by the tissue and its microenvironment. Inflammation and pathological conditions can change the steady state of ILCs in tissue directing their role in the course of the disease (36). Specific alterations in the cytokine concentrations in the tumor microenvironment result in different changes in ILC composition. In the tumor microenvironment, ILC composition and functions are heavily influenced by the different cytokines, immune cells and immunosuppressive milieu which can lead to their trans differentiation into other subsets leading to a pro or anti-tumor role (34, 38, 50–55). Here, we summarize the findings from numerous murine and human studies describing the plasticity of ILC subsets in homeostasis and pathological conditions with a focus on ILCs in the tumor microenvironment.

## NK 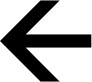 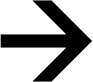 ILC1 Plasticity

ILC1 and NK cells share many characteristics as they both produce IFN-γ, express the transcription factor T-bet (not expressed in NK cells at the same level) and require interleukin-15 (IL-15) for their development (35, 56). Although NK cells and ILC1s express similar genes, they are functionally and developmentally different.

A very early study describes the influence of IL-15 on NK-like cells in murine cancer models. Dadi et al. describe an innate-like subtype of TCR^-^NK1.1^+^NKp46^+^ CD49a^hi^ cells that express high amounts of GzmB and share transcriptomic profiles of ILC1 and conventional NK cells. Importantly, this ILC1-like population did not express the ILC marker CD127. Other typical ILC1 cytokines like IFN-γ and TNF-α were expressed at lower levels compared to typical ILC1 cells. The population occurs in cancerous lesions and exhibit innate cytotoxicity toward tumor cells when stimulated with IL-15 (57). Both murine and human studies point to the influence of the cytokine milieu along with other environmental signals on regulation of NK cell plasticity and how conversion of cytotoxic NK cells into ILC1s might support tumor growth. It remains to be seen whether ILC1 can transdifferentiate into NK cells under physiological conditions.

In humans, the local environmental signals induce several subsets in between NK and ILC1 cells. An ILC1 subset was reported in tonsil and gut epithelium which expressed alpha E beta 7 (aeb7) integrin (CD103), NKp44 and secreted IFN-γ upon IL-12 and IL-15 stimulation (56). Human NKp44^+^CD103^+^ intraepithelial (ie) ILC1 cells lack CD127, are cytotoxic and express T-bet and EOMES showing their resemblance to NK cells. Interestingly, ieILC1s were detected at higher frequencies within the small intestine of patients with Crohn’s disease pointing towards their role in disease pathogenesis (56). The intraepithelial ILC1s were also identified by mass cytometry in the human intestine (44). The mouse counterpart of intraepithelial ILC1s express CD160 and Nfil3 (Nuclear Factor, Interleukin 3 Regulated) -TBX21 (T-Box Transcription Factor 21) encoded transcription factors. Another study in patients with head and neck cancer used scRNA-seq to identify trans-differentiation of conventional NK cells into CD49a^+^CD103^+^ ieILC1-like cells and a NR4A2 (Nuclear Receptor Subfamily 4 Group A Member 2) -expressing CD49a^–^ subset, of which the first one provided a strong anti-tumor immunity and the second showed only low capacity to control tumor growth. This was dependent on IL-15 and transforming growth factor (TGF)-β signaling, both important cytokines driving NK-ILC1 plasticity (58).

Gao et al. studied the role of TGF-β in methycholantrene (MCA) induced tumor models in mice (14). TGF-β plays an important role in the tumor environment and regulates the conversion of NK cells (CD49a^-^CD49^+^EOMES^+^) into ILC1 (CD49a^+^CD49b^-^Eomes^int^) or intermediate (intILC1, CD49a^+^CD49b^+^EOMES^+^) cells (14). Interestingly, the ILC1 and intILC1 cells were not able to control growth of tumors or metastasis as opposed to the tumor suppressive NK cells (14). This transition is negatively regulated by SMAD4 (mothers against decapentaplegic homolog 4), through TGFBR1 receptor in SMAD4-deficient mice (59). Lack of SMAD4 did not affect ILC1 differentiation, however, NK cells in these mice transitioned into ILC1 like cells. These ex-NK-ILC-1 like cells were unable to control tumor growth as opposed to NK cells influenced by TGF-β (59) and had reduced production of IFN-γ.

We have recently performed a comprehensive transcriptomic profile of human ILCs from liver in HCC patients and have shown the transition of cytotoxic NK cells in the non-tumor liver tissue into non cytotoxic ILC-1 like cells in the tumor infiltrating ILCs (49). We detected higher levels of TGF-β, IL-10, and IL-8 in the liver tumor supernatants in line with the transcriptomic profile of the HCC tumors and non-tumor tissue (49). Differentiation of NK cells into ILC1s has also been shown in mice with non-alcoholic fatty liver disease (60) as well as in infection with *toxoplasma gondii* where it was shown that IL-12 plays in role in the conversion of NK cells into ILC1 cells by suppression of EOMES and upregulation of T-bet expression (61).

These studies showed that exposure to cytokines like TGF-β in the tumor microenvironment can regulate the plasticity of ILC1-NK cells which can affect the biological endpoint such as allowing the tumors to escape immune surveillance. Additionally, mass cytometry by CYTOF showed *in vitro* culture of human NK cells with TGF-β and IL-15 converts human NK cells into ILC1s with a progressive change in the cell surface and activation markers such as acquisition of tissue residency markers like CD69, CD9 and CD49a (62).

Another study used MCA induced tumor models and NKp46^+^ ILC-deficient mice to identify the role of NKp46-dependent ILCs in tumor development. Similarly, to the previous study, the infiltration of ILC1s was correlated with lower anti-tumor immune response (63).

ScRNAseq has led to the identification of small changes in the transcriptomic profile of ILCs and has identified tumor specific subsets of ILCs. In colorectal cancer (CRC), tissue specific ILC1-like populations were identified, be unique for tumor tissue, but had some overlap with an ILC1like subset in the blood of CRC patients. This ILC1-like population expressed significantly more SLAMF1 compared to healthy mucosa. Low SLAMF1 expression was associated with poor survival in CRC and rectum cancer which identified SLAMF1 as a potential biomarker and SLAMF1^+^ ILCs as a population with anti-tumor effect (64).

## ILC3 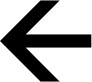 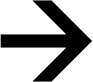 NK Plasticity

ILC3s have a significant influence on anti-tumor immunity. Specifically, the extensive interaction with the adaptive immunity makes ILC3s highly important for an effective immune response. IL1β and CCL20 have been found to be important cytokines which lead to recruitment of ILC3s to the tumor site. ILC3s finally activate CD4^+^ and CD8^+^ T cells and induce a strong anti-tumor immunity. Cisplatin therapy further increased ILC3 concentration as well as ILC2 and NK cells. In this study, no plasticity between ILCs could be analyzed but rather direct infiltration of ILCs into the tumor (65).

Recent studies have proposed that human ILC3 can transdifferentiate into NK cells although they are of different lineages. Fully mature human ILC3s can convert into CD94^+^T-bet^+^EOMES^+ ^NK cells as facilitated by pro inflammatory cytokines IL-12 and IL-15 (66). Additionally, AHR transcription factor inhibits ILC3 differentiation into NK cells in the presence of IL-1β (67). Although the ILC3-NK cell trans-differentiation has not been shown *in vivo*, one can speculate that in response to environmental stimuli, changes in the AHR/IL-1R can lead to expansion of ILC3 cells or conversely change them to NK cells leading to different pathological outcomes as dictated by the inflammatory tissue.

In human ovarian cancer, a population of regulatory CD56^+^CD3^-^ ILCs were found, which showed characteristics of NK cells but produced IL-22 without showing other specific markers of ILC3s (68).

We have also shown that NK-like cells in the non-tumor tissue of HCC patients convert to ILC1 and ILC2 in the tumor. Trajectory analysis from scRNA-seq identified ILC3s to be the intermediate population in this transition from NK to ILC1 or ILC2 cells. A reversible plasticity could not be observed (49).

## ILC3 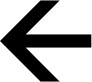 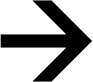 ILC1 Plasticity

Plasticity of ILC3s and their conversion into ILC1 cells was initially shown in mice (69). Fate mapping studies using RORc mice showed that NKp46^+^ILC3 cells can convert into NK1.1^+^ ILC1 (ex-ILC3 cells) with a concomitant decrease in RORγt and increase in T-bet and Notch signaling (69, 70). The trans-differentiation of ILC3 into ILC1s in mice was prevented in TBX21^-/-^ mice (47, 70, 71). This was also shown in human where RORγt^+^ fetal gut NKp44^+^ILC3 subset upon culturing with IL-2 and IL-12 lost their NKp44 expression, downregulated RORγ and c-kit expression and differentiated into ILC1 cells. This was accompanied by upregulation of *TBX21* and IFN-γ production (10). Interestingly, in intestinal biopsies of patients with Crohn’s disease (CD) and ulcerative colitis (UC), there was a change in the composition of the ILC subsets. In patients with Crohn’s disease and a Th1 inflammatory phenotype, a reduction in ILC3 frequency in inflamed ileal resections was seen as opposed to higher frequency of ILC1 in patients with no inflammation (10). A reduction of NKp44^+^ ILC3s in both diseases was detected which was parallel to an increase in ILC1 and ILC2 subsets showing the impact of the inflammation on ILC composition and their plasticity (13).

Further work on the plasticity of ILC1 and ILC3 cells has focused on identification of intermediate cells in between the two ILC subsets. RNA analysis of human tonsil has identified several subpopulations between CD103^+^ ILC1 and ILC3 cells which were distinct from conventional NK cells (46). These subsets showed graded expression of ILC1 and ILC3-specific markers such as CD103, CCR6 reflecting the plasticity between the different ILC subsets. RNA velocity showed one ILC3-ILC1 intermediate population which had more an ILC1 like phenotype and was also detected in the human intestinal mucosa (46). We have shown that in patients with HCC, ILC3 cells in the non-tumor can transdifferentiate into ILC1s and ILC2s in the tumor liver tissue (49). In melanoma tumors the presence of NKp46(+) LTi cells induced upregulation of adhesion molecules in the tumor vasculature and resulted in more leukocyte invasion. This was mediated by IL-12 (72).

The transcriptional regulation of the plasticity of ILC3-ILC1 has shown to be complex involving Aiolos, Ikaros as well as BCL-6 and MAF transcription factors by controlling of IL-12 and IL-23-induced signaling (73). The differentiation of ILC3 to ILC1 is a reversible process where human CD127^+^ILC1 can change to ILC3 when cultured with IL-2, IL-23, and IL-1β, a process that was RORγt dependent and enhanced in the presence of retinoic acid (RA). This change was also confirmed *in vivo* in NOD SCID *IL2gc*^−/−^ (NSG) mice that were engrafted with human immune cells and was reversible (45).

MHC class II expression on ILC3 regulates T cells responses to commensal bacteria and in the intestine of pediatric patients with intestinal bowel disease (IBD), MHC class II expression on ILC3 was significantly reduced, but not on any other immune cells as compared to non-IBD controls (74). For example, HLA-DR^+^ -ILC3 are not only concentrated in lung draining lymphoid tissues of healthy mice and humans, but also can limit type 2 mediated allergic responses (75).Recently, ILC3s were shown to secrete a tissue protective growth factor (HB-EGF) in the intestinal mucosa which protects the intestinal epithelium from TNF induced death and reduces disease severity in acute and chronic intestinal inflammation (76). Therefore, ILC3 cells can balance the tissue they reside in under homeostasis or contribute to progression of inflammation.

Another ILC subset of regulatory ILCs (ILCreg) which produce IL-10 have also been described (77). In colorectal cancer, ILC3s were converted into ILCregs by TGF-β (78). Nevertheless, another study could not find the ILCreg subset in several mouse models but described an ILC2 population which produced IL-10 and contributed to intestinal inflammation (79). The authors speculate that microbiome composition might result in different subsets of ILCs (79).

ILC3s lacking the natural cytotoxicity receptor (NCR^-^ ILC3) were shown to promote development of murine HCC in response to IL-23, where IL-23 induced the differentiation of ILC1s to ILC3s (80) similar to what has been shown previously where IL-23 could drive the differentiation of human ILC1 into ILC3 cells (45).

Overall, these studies identify heterogeneity of ILC3s in pathological conditions and specifically in cancer. The dominating ILC3 population is influenced by the cytokine environment which has significant influence on the pro- or anti-tumor function of ILC3s.

## ILC2 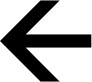 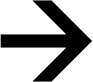 ILC1 Plasticity

In both mice and human, IL-25 and IL-33 are the main cytokines that trigger ILC2 activation and induction and release of IL-13, IL-5 and IL-9 cytokines (28, 81, 82). ILC2s have been shown to differentiate into IFN-γ producing ILC1 -like cells both *in vivo* and *in vitro*. When ILC2 cells from peripheral blood were cultured with IL-1β and IL-12, there was an increase in IFN-γ production as well as upregulation of T-bet expression (83). Furthermore, upregulation of IL-33 and IL-25 receptors on ILC2s was enhanced by IL-1β which potentiated the responses to these cytokines (<ξ>83). The biological significance of the plasticity of the circulating ILC2s to switch to IFN-γ secreting cells enables the immune system to respond rapidly to different pathological conditions. This could have both beneficial or harmful effects depending on the balance of the cytokines and effector mechanism. As an example, IFN-γ^+^-ILC2 were detected in intestine of patients with Crohn’s disease as well as in lung tissues from patients with chronic obstructive pulmonary disease (COPD) (12, 84, 85). ILC1 can be converted back to ILC2 by IL-4, which is produced by basophils, eosinophils and ILC2 cells as part of the inflammatory Th2 responses (85).

HCC is an inflammatory type of cancer and ILC2s seem to play an important role in this disease. We have shown that ILC2s are the main population in the tumor from HCC patients and are derived from intermediate ILC3s or ILC1s as shown by trajectory analysis of the transcriptomic analysis of these cells both in adjacent liver and tumor tissue (49). High ILC2 frequency was associated with better overall survival (49). In contrast, in a murine model of HCC, ILC2s were found to be detrimental for tumor growth in HCC. Notch signaling could convert ILC2s into inflammatory ILC2s which had characteristics of ILC2/ILC3 cells by expressing IL-13 and IL-17 (86). The same group revealed a significant increase in the overall ILC2 frequency in HCC tissue relative to the paired non tumor liver tissues. These ILC2s were KLRG1 negative and recruited immunosuppressive neutrophils *via* CXCL2 expression which finally resulted in poor anti-tumor immune response and poor survival (87). However, a clear trans differentiation could not be revealed, indicating active proliferation and self-renewal of hepatic ILC2s in the HCC microenvironment (87).

ILC2s have also been shown to have an anti-tumor role in pancreatic cancer. IL-33 was enriched in pancreatic cancer and tumor-infiltrating ILC2s were activated by IL-33 which resulted in activation of CD8^+^ T cells and finally a strong anti-tumor immune response. This could be further enhanced by treatment with checkpoint inhibitor anti-PD1 (88). In our studies we have shown that IL-33 is increased in HCC tumors as well and high ILC2 concentration in the tumor was associated with better survival (49). In HCC, we found that ILC2s are derived from ILC3s and ILC1s *via* trans differentiation from non-tumor tissue to tumor tissue as affected by the tumor microenvironment (49).

## ILC2 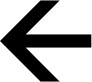 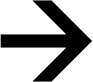 ILC3 Plasticity

Recent studies add to the complexity of ILC plasticity, in particular ILC2 cells where ILC2s can be classified into two groups based on their reaction to IL-33 and IL-25 (28, 89). Natural ILC2s (nILC2) mainly reside in barrier tissues, respond to IL-33, have high levels of IL-33 receptor (ST2+) and are mainly tissue resident playing important roles in immune protection as well as tissue repair. However, inflammatory ILC2s (iILC2) are circulating, respond to IL-25 and can express RORγt and IL-17 and develop into nILC2 or ILC-3 like cells depending in response to infection or inflammation in the tissue (90). Inflammatory ILC2s (iILC2) have been shown in infection settings such as protection against *Candida albicans* (90, 91).

Interestingly, human ILC2s are the only subset of ILCs that have a heterogenous expression of SCF receptor c-kit (CD117). ILC3 cells express CD117 but have no expression of ILC2 markers and ILC1 express neither ILC1 nor ILC3 markers. ILC2s from nasal tissue, blood, tonsils, and intestine have variable expression of c-kit (41, 44). ILC2 cells with high c-kit expression (c-kit^hi^) also express RORγt and secrete IL-17, which are shared characteristics of ILC3 cells and are found mainly in skin, thymus, blood, tonsil and intestine. ILC2-c-kit^lo^ cells mainly produce type 2 cytokines and are found in blood, tonsil and intestine. The dynamic expression of c-kit on ILC2s shows to be a regulator of plasticity in these cells enabling them to respond to pathological settings. Culturing peripheral blood ILC2 subsets (c-kit^hi/lo^) in ILC3 conditioning cytokines showed that ILC2c-kit^hi^ cells showed ILC3 characteristics and expressed CCR6 (present on ILC3s) and produced IL17A (92). Interestingly, TGF-β promotes the conversion of c-kit-ILC2s into RORγt expressing cells by inducing c-kit and CCR-6 and downregulating GATA-3 in these cells. The plasticity potential of ILC2 to convert into ILC3 cells under conditions where higher levels of TGF-β is present can contribute to physiological settings with IL-17 mediated pathways such as in skin lesions of patients with psoriasis (93) or under other inflammatory conditions. In nasal polyps of patients with cystic fibrosis, IL-17 producing CD117^+^ Nkp44 ILC3 cells were detected at high frequency and that these cells are derived from ILC2s by IL-1β, IL-23 and TGF−β derived from the nasal epithelial cells (94). Thus, the plastic nature of ILC2 and their differentiation into IL-17 secreting ILC3-like cells, enables these cells to effectively respond to a diverse range of environmental stimuli depending on the infection or inflammation. On the other, we found that ILC3s can convert into ILC2s through an intermediated state of c-KIT^+^ ILCs in patients with liver cancer. This was mediated by a cytokine gradient from non-tumor to tumor tissue involving among others the cytokines IL-33, IL-4 and IL-1β. This points out that ILC2-3 plasticity is highly dependent on individual environments, leading to different compositions and function of ILC2s and ILC3s in the specific setting.

## Summary

**Figure 1:**
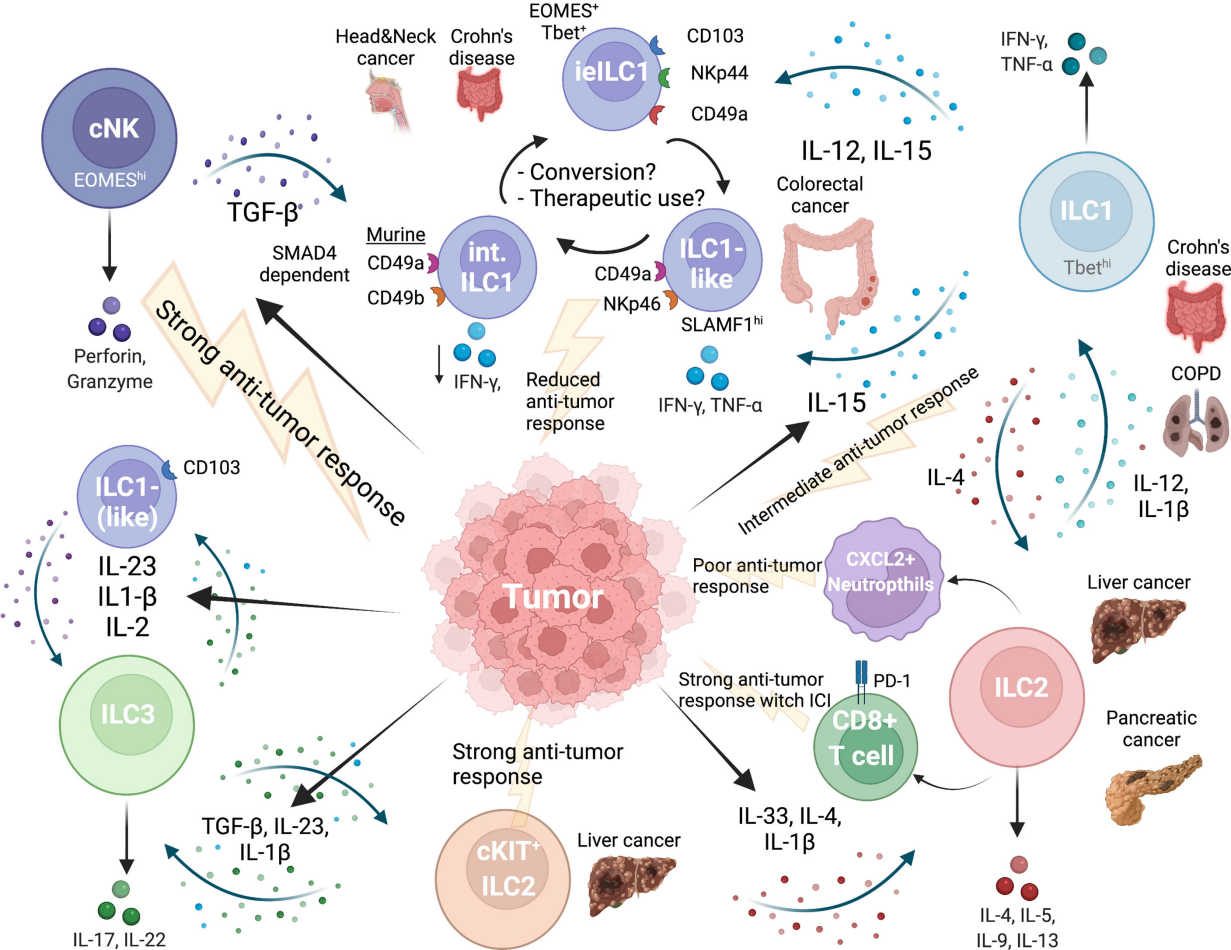
Network of ILC plasticity in cancer. Tumor microenvironment influences all ILC subgroups. EOMES^hi^ NK cells provide the strongest anti-tumor immune response. TGFβ released by tumor cells induces conversion of NK cells into intermediate or ILC1-like cells and finally into ILC1s, which are less cytotoxic and provide a weaker anti-tumor response. Intraepithelial ILC1 have been specifically described in the gut and express CD103. Another intermediate ILC1 population can be found in colorectal cancer (CRC), is NKp46 positive and regulated by SLAMF1, which is associated with anti-tumor immunity in CRC. An intermediate NK-ILC1 population shows marker of both subgroups (CD49a^+^CD49b^+)^ in mice and is associated with reduced anti-tumor immune response. A direct NK-ILC2 plasticity has not been described so far. ILC2s and ILC1s can undergo plasticity depending on the cytokine milieu. In pancreatic cancer and liver cancer ILC2s were associated with a strong anti-tumor response, also when immune checkpoint inhibitor treatment (ICI) was used. In mice, ILC2s activated CXCL2^+^ neutrophils which were immunosuppressive. ILC3s are high in frequency in liver and intestine and can are a highly plastic population. ILC3s can transition back and forth into ILC1 and can differentiate into ILC2 in liver cancer along a cytokine gradient dictated by the secretion of cytokines from the tumor. Created with BioRender.com

Numerous studies have advanced our current knowledge of ILCs and shown their significant and pivotal role not only in tissue homeostasis but also in inflammatory and pathological conditions. Specifically, in tumors, ILC plasticity is heavily influenced by the type of tumor as well as the tumor microenvironment facilitating their role in tumor progression or anti-tumor immunity. The cytokines in inflammatory and cancerous tissue influence ILCs which can act either directly or indirectly on tumor immunity. The future challenges will be to harness the plasticity of ILC subsets in tumors which has important implications in development of promising immunotherapeutic approaches. Here, we have presented a comprehensive overview of the studies on plastic transitions of ILCs in the tumor environment (**Figure 1**) to help understand their complex network and to potentially use them as therapeutic targets.

## Author Contributions

BH: literature research, writing, review of the manuscript. FK: literature research, writing, review of the manuscript. All authors contributed to the article and approved the submitted version.

## Funding

This project was supported by the Intramural Research Program of the NIH, NCI (ZIA BC 011345).

## Conflict of Interest

The authors declare that the research was conducted in the absence of any commercial or financial relationships that could be construed as a potential conflict of interest.

## Publisher’s Note

All claims expressed in this article are solely those of the authors and do not necessarily represent those of their affiliated organizations, or those of the publisher, the editors and the reviewers. Any product that may be evaluated in this article, or claim that may be made by its manufacturer, is not guaranteed or endorsed by the publisher.
